# The past, present and future of the International Journal of Behavioral Nutrition and Physical Activity

**DOI:** 10.1186/1479-5868-3-6

**Published:** 2006-03-28

**Authors:** Johannes Brug, Simone French, Bente Wold

**Affiliations:** 1 Dept. of Public Health, Erasmus University Medical Center, Rotterdam, The Netherlands; 2 Division of Epidemiology and Community Health, University of Minnesota, Minnesota, USA; 3 Dept. of Education and Health Promotion, University of Bergen, Bergen, Norway

## The past

The International Journal of Behavioral Nutrition and Physical Activity (IJBNPA) has celebrated its third birthday. In February 2004 the IJBNPA (IJBNPA) was launched with the first official IJBNPA publication, an editorial of the founding editors-in-chief of the journal, Simone French and Tony Worsley. This official start was the result of more than six months of careful planning and preparations following the first annual meeting of the International Society of Behavioral Nutrition and Physical Activity in Seattle in June 2003 and involving the Executive and Communications Committees of the society.

## The present

The journal is now in its fourth year. There is a steady growth in the number of papers that are submitted to the journal (2003: 29; 2004: 66; 2005: 97). The journal is dedicated to only publish high-quality papers on innovative research on physical activity and eating behaviors. The number of papers that meet our strict quality and innovation criteria is limited, which is reflected by the number of papers that actually gets published (2004: 17; 2005:18).

Because IJBNPA is an electronic journal, authors learn very quickly whether their paper will be published in the IJBNPA, and this swift review and publication process means that important research results, new theoretical insights, or important commentaries on emerging behavioral nutrition and physical activity issues can be shared with the relevant scientific and practice communities in a timely manner. The average time from initial submission to publication is 161 days.

The papers that are published by the IJBNPA garner attention. According to the tracking information that is provided by the IJBNPA publisher, BioMed Central, in January 2006 ten of the 35 papers published in the IJBNPA were 'highly accessed' (accessed more than 1,000 times per month). Nine papers have been accessed more than 4,000 times, and the most frequently accessed paper was accessed more than 30,000 times over all [1]. These impressive data illustrate the importance of IJBNPA being an 'open access' journal. Despite IJBNPA's recent inception, papers published in IJBNPA are already beginning to be cited in other journals.

The papers published by the IJBNPA are mainly original empirical studies on important behavioral nutrition and physical activity issues. However, commentary papers are also published. For example, three ' theory debate' papers were recently published that discussed the strengths and weaknesses of behavioral theories applied in behavioral nutrition and physical activity research and practice [2-4].

In 2005, the IJBNPA editorial board was expanded to 21 members from a wider range of countries (10 countries represented) and a further expansion is underway. IJBNPA is supported by a large and diverse group of peer-reviewers. These researchers who are willing to allocate their valuable time to provide critical comments to papers submitted to IJBNPA form the real backbone of the journal. Peer-review is an important basis for scientific quality control and progress, including that of IJBNPA.

The journal is now indexed in PubMed and Embase, and indexing in Psychinfo is underway.

## The future

In their 2004 kick-off editorial French and Worsley clearly presented the goals of IJBNPA [5] to "*encourage and disseminate novel and innovative research on physical activity and eating behaviors*", with an "*international focus*" to "*to redress the current narrow focus of models and interventions that have been developed and evaluated in homogeneous settings in the western industrialized world*". IJBNPA aims to "*include multiple levels of analysis, including populations, groups and individuals*" and "*include epidemiology, and behavioral, theoretical and measurement research areas*", while maintaining "*methodological rigor and high standards of scholarship*".

Thanks to the authors, peer-reviewers, editorial board members, ISBNPA members, and others who have contributed to the journal, the IJBNPA has begun to realize these important goals. But we are not there yet; the journal needs to be further strengthened.

First, the number of high-quality papers published in IJBNPA needs to increase. At this stage, all ISBNPA members are called to submit their innovative and high-quality work to IJBNPA and to encourage their colleagues to do so.

One important criterion considered by many when deciding where to submit their most interesting research papers is a journal's 'Impact Factor' (IF). The (in)famous score is based on how often papers in a journal are cited by others. Journals must meet several criteria prior to receiving an IF. Because it is so new, the IJBNPA does not yet have an IF. The editors are working with the IJBNPA publishers to develop the journal so that an IF can be given to the journal. So your high-quality papers are needed more than ever to continue to build a stronger IJBNPA with a high IF. Your support is also needed through encouraging your colleagues to submit their highest quality research papers to the IJBNPA and to frequently cite articles published in the IJBNPA in their own published papers.

The Editors of the IJBNPA have been energized by the quality and quantity of interesting and innovative research published in the journal to date, and by the support for the journal from the nutrition and physical activity behavioral research community. Thank you for sharing your best work with the journal and for actively participating in the development of this exciting new research area. We look forward to working together to move the field forward into the future.
